# Digital Health Literacy and Web-Based Information-Seeking Behaviors of University Students in Germany During the COVID-19 Pandemic: Cross-sectional Survey Study

**DOI:** 10.2196/24097

**Published:** 2021-01-15

**Authors:** Kevin Dadaczynski, Orkan Okan, Melanie Messer, Angela Y M Leung, Rafaela Rosário, Emily Darlington, Katharina Rathmann

**Affiliations:** 1 Department of Nursing and Health Sciences Fulda University of Applied Sciences Fulda Germany; 2 Centre for Applied Health Science Leuphana University Lueneburg Lueneburg Germany; 3 Interdisciplinary Centre for Health Literacy Research Faculty of Educational Science Bielefeld University Bielefeld Germany; 4 Section "External Lecturer" APOLLON University of Applied Sciences Bremen Germany; 5 Centre for Gerontological Nursing, World Health Organization Collaborating Centre for Community Health Services School of Nursing Hong Kong Polytechnic University, Hong Kong SAR Hong Kong China; 6 School of Nursing University of Minho Braga Portugal; 7 Health Systemic Process EA 4129 Research Unit (P2S) University of Lyon University Claude Bernard of Lyon 1 Lyon France

**Keywords:** digital health, literacy, infodemic, health information, behaviour, coronavirus, COVID-19, university student, student, infodemiology

## Abstract

**Background:**

Digital communication technologies are playing an important role in the health communication strategies of governments and public health authorities during the COVID-19 pandemic. The internet and social media have become important sources of health-related information on COVID-19 and on protective behaviors. In addition, the COVID-19 infodemic is spreading faster than the coronavirus itself, which interferes with governmental health-related communication efforts. This jeopardizes national public health containment strategies. Therefore, digital health literacy is a key competence to navigate web-based COVID-19–related information and service environments.

**Objective:**

This study aimed to investigate university students’ digital health literacy and web-based information-seeking behaviors during the early stages of the COVID-19 pandemic in Germany.

**Methods:**

A cross-sectional study among 14,916 university students aged ≥18 years from 130 universities across all 16 federal states of Germany was conducted using a web-based survey. Along with sociodemographic characteristics (sex, age, subjective social status), the measures included five subscales from the Digital Health Literacy Instrument (DHLI), which was adapted to the specific context of the COVID-19 pandemic. Web-based information-seeking behavior was investigated by examining the web-based sources used by university students and the topics that the students searched for in connection with COVID-19. Data were analyzed using univariate and bivariate analyses.

**Results:**

Across digital health literacy dimensions, the greatest difficulties could be found for assessing the reliability of health-related information (5964/14,103, 42.3%) and the ability to determine whether the information was written with a commercial interest (5489/14,097, 38.9%). Moreover, the respondents indicated that they most frequently have problems finding the information they are looking for (4282/14,098, 30.4%). When stratified according to sociodemographic characteristics, significant differences were found, with female university students reporting a lower DHLI for the dimensions of “information searching” and “evaluating reliability.” Search engines, news portals, and websites of public bodies were most often used by the respondents as sources to search for information on COVID-19 and related issues. Female students were found to use social media and health portals more frequently, while male students used Wikipedia and other web-based encyclopedias as well as YouTube more often. The use of social media was associated with a low ability to critically evaluate information, while the opposite was observed for the use of public websites.

**Conclusions:**

Although digital health literacy is well developed in university students, a significant proportion of students still face difficulties with certain abilities to evaluate information. There is a need to strengthen the digital health literacy capacities of university students using tailored interventions. Improving the quality of health-related information on the internet is also key.

## Introduction

Shortly after the outbreak of SARS-CoV-2 and the associated disease, COVID-19, were first reported [1], it was declared a pandemic [2,3] by the World Health Organization. When the first case of COVID-19 was reported in Germany on January 27, 2020, the government responded immediately by launching an unprecedented nationwide emergency response plan that focused on four pillars: prevention, detection, containment, and treatment [4]. In addition to the National Pandemic Plan [5] and to health care and medical interventions [4], the government endorsed a public health communication strategy that was supported by all health agencies and public health bodies on national and local levels [6-9]. This communication strategy involved public broadcasting agencies, which launched web-based media campaigns, including daily nationwide podcasts. The underlying objective of this approach was to provide citizens with the necessary information on COVID-19 and how it affected people’s health [4,10-13]. Within a short period of time, a massive amount of web-based health-related information on COVID-19 became available on issues such as protective behaviors, preventive measures, treatment options, dashboard statistics, the latest scientific insights, and various safety recommendations [14-16]. It has now become clear that this pandemic has been accompanied by an “infodemic”—an overabundance of valid and invalid health information on COVID-19 [17,18]. By means of digital communication technologies, especially the internet and social media, the COVID-19 infodemic is spreading faster than the coronavirus itself, which interferes with governmental health communication efforts and jeopardizes national public health containment strategies.

Altogether, this situation creates a complex information environment that requires people to be able to access, navigate, understand, use, and critically evaluate information and services in ways that support healthy and protective behaviors in the time of the COVID-19 pandemic. Therefore, health literacy, which is the ability to find, understand, and evaluate health information and apply it in daily decision-making and health behavior [19], is of utmost importance during the current pandemic [14]. Digital health literacy applies this understanding of health literacy to digital contexts and environments [20], and it has become a core competence and necessity for navigating web-based information and health service environments within the realm of the COVID-19 pandemic and the associated infodemic [21]. However, in Germany, it has been shown that more than half of the population has limited health literacy; therefore, people report difficulties in dealing with health-related information [22]. A recent study conducted in Germany on health literacy in relation to information regarding COVID-19 resulted in similar findings [23]. People particularly have difficulty assessing the trustworthiness of media information on COVID-19 and its associated health problems. In addition, people with limited health literacy are more likely to be confused due to the massive amounts of information available in the media and on the internet [23]. Information is a carrier of important health knowledge to contain the virus and empower citizens to demonstrate health literacy [16,24]; the pandemic has placed increased demand on the general population to find information relevant to them and critically reflect on this information, as well as to transfer information into their everyday life and practices.

This issue is particularly critical for university students, who consist of a significant proportion of young adults in Germany. University students comprise the population that primarily uses digital technologies and web-based health information [25,26]. Although it can be noted that students have not been the primary focus of research since the beginning of the pandemic, a recent study with over 5400 medical students from Vietnam revealed that higher levels of health literacy were associated with less fear of COVID-19 [27]. Therefore, health literacy is a critical intervention target, especially since fear is one of the toxic outcomes that result from an infodemic [17,28]. The aim of this study is to investigate the digital health literacy and web-based information-seeking behaviors among university students in Germany during the first wave of the COVID-19 pandemic, particularly during university closures. This study is informed by the conceptual model of health literacy as presented by Sørensen and colleagues [19] and the model of digital health literacy as proposed by van der Vaart and Drossaert [20]. The assumptions in both models are that personal and environmental determinants influence an individual’s capacity regarding various dimensions of personal information management, which include informing health decisions and behaviors that are beneficial for health. Our study focuses on personal and environmental determinants, personal information management, and behavioral aspects. In this context, the following research questions were addressed:

- What are the levels of COVID-19–related digital health literacy in German university students stratified by social, economic, and geographical indicators?
- Which sources of web-based information are used and which topics are searched for in the context of COVID-19 by German university students?
- Can differences be identified between students with regard to health literacy, the sources used for information searching, and the topics addressed in relation to COVID-19?

## Methods

### Study Design and Participants

**Figure 1:**
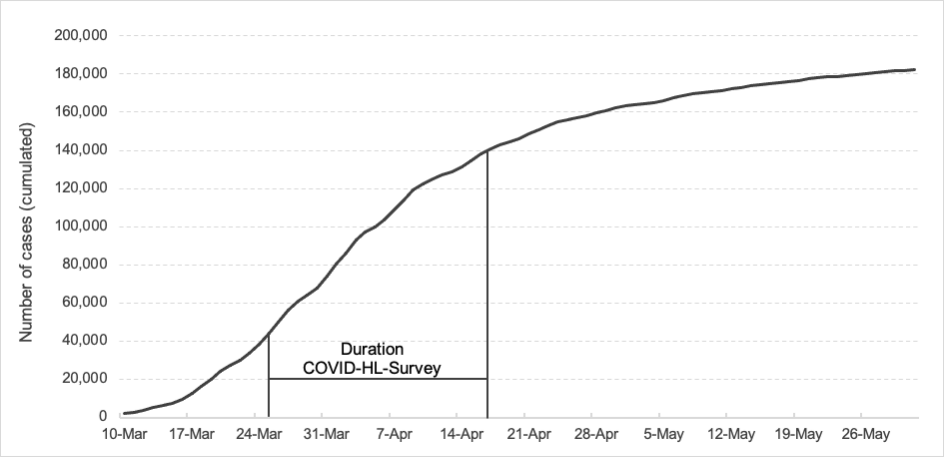
Confirmed cumulative cases of COVID-19 in Germany from March to May 2020 (source: RKI COVID-19 Dashboard [30]).

A national cross-sectional web-based survey was conducted including a nonrandomized sample (convenience sample) of German university students. To address as many university students as possible, all private and state universities (ie, 392 universities containing 2.9 million students [29]) were invited to participate in the study by email. A reminder was sent two weeks after the survey started. The presidencies of all the universities and the deaneries of all faculties were contacted and asked to forward an invitation letter to their students. University students enrolled at a private or state university were eligible to participate in this study. To increase the homogeneity of the sample, respondents were initially asked to indicate their current status. Those who indicated that they were not currently enrolled as students at a German university were excluded from the data set (n=245). The duration of the study was 3 weeks, and it took place from March 25 to April 17, 2020. Within the 3 weeks during which the survey was implemented, the number of confirmed COVID-19 cases in Germany increased from 44,175 to 141,016 (Figure 1 [30]). The survey was administered electronically using the Enterprise Feedback Suite survey tool (Questback) [31]. Participation was voluntary, and anonymity was ensured. Upon entering the web-based survey site, participants were presented with information regarding the background and the aims of the study. After checking a consent box at the bottom of the page, participants were directed to the questionnaire. Our study was approved by the Bielefeld University ethics committee (No. EUB 2020-053).

### Measures

Sociodemographic information included sex (male, female, diverse), age, study course (bachelor’s degree, master’s degree, other), and subjective social status (SSS). Age was measured in absolute numbers, and based on an analysis of the distribution, four categories were created (≤20 years, 21-23 years, 24-26 years, and ≥27 years). Social status was assessed using the German version of the MacArthur Scale, which includes a ladder with 10 steps [32]. Respondents were asked to position themselves at the step that best reflected their status in the social hierarchy, with higher values indicating a higher social status. According to previous studies, respondents were categorized into three groups: low SSS (1-4), medium SSS (5-7), and high SSS (8-10) [33].

Digital health literacy was evaluated using five of the seven subscales from the validated Digital Health Literacy Instrument (DHLI) [20], each including three items to be answered on a 4-point scale (eg, 1, very difficult; 4, very easy). The DHLI was adapted to the context of the COVID-19 pandemic (eg, “When you search the Internet for information on the coronavirus or related topics, how easy or difficult is it for you to…”). The five subscales include (1) searching the web for information on COVID-19, (2) adding self-generated content on COVID-19, (3) evaluating the reliability of COVID-19–related information, (4) determining personal relevance of COVID-19–related information, and (5) protecting privacy on the internet. The internal consistency (Cronbach α) of the first four subscales was acceptable to good (.70<α<.83). Due to low reliability (α=.46), scaling was omitted for the protecting privacy subscale.

The section about web-based information-seeking behaviors focused on the sources that were used to receive web-based health information about COVID-19 and related topics. The respondents were presented with a 10-item list of different web-based sources (eg, search engines, websites of public health bodies, government agencies, and social media providers), in which the frequency of their use could be rated on a 5-point scale (0, don't know; 4, often) [34]. Students were also asked to indicate the specific topics they searched for in the context of COVID-19. The assessment was based on a self-developed list of 9 topics (eg, current spread of COVID-19, symptoms of COVID-19, measures to protect against infection, dealing with psychological stress caused by the COVID-19 pandemic), using yes or no answers.

An overview of all items and scales used for this paper can be found in Multimedia Appendix 1. The entire questionnaire is available on request from the first authors.

### Statistical Analysis

To control for the selection bias caused by a convenience sampling procedure, we used weighting to adjust the sample distribution to the characteristics of the general population of German university students. Based on the data provided by the Federal Statistical Office via the GENESIS database [35], the data could be weighted for gender and desired study degree. In the first step, all data on digital health literacy and information-seeking behavior were analyzed descriptively. Subsequently, bivariate analyses were conducted by cross-tabulating the two levels of digital health literacy (limited vs sufficient) with sociodemographic characteristics using chi-square tests. For this purpose, all DHLI subscales (except “protecting privacy”) were dichotomized using median splits. Due to the low internal consistency for the dimension “protecting privacy” and the fact that two subscales from the original DHLI instrument were not used, we also refrained from calculating an overall mean value, as done by Van der Vaart and Drossaert [20]. For all analyses, *P* values <.05 were considered statistically significant. However, due to the large sample size, the strength of the association was determined using the Cramer index (Cramer V). The Cramer V is a normalized version of the chi square statistic test for nominal scaled variables. According to Cohen [36], the strength of each association was interpreted as an effect size measure using the following conventions: ≥0.1 (small), ≥0.3 (medium), ≥0.5 (large). In further analyses, chi-square tests were also performed for the levels of digital health literacy and the topics searched for with regard to COVID-19. Finally, to analyze differences between the levels of digital health literacy and the sources used to search for COVID-19–related information, *t* tests for independent samples were conducted. Cohen *d* was used as an effect size measure by applying the following conventions: ≥0.2 (small), ≥0.5 (medium), ≥0.8 (large) [36].

## Results

**Figure 2:**
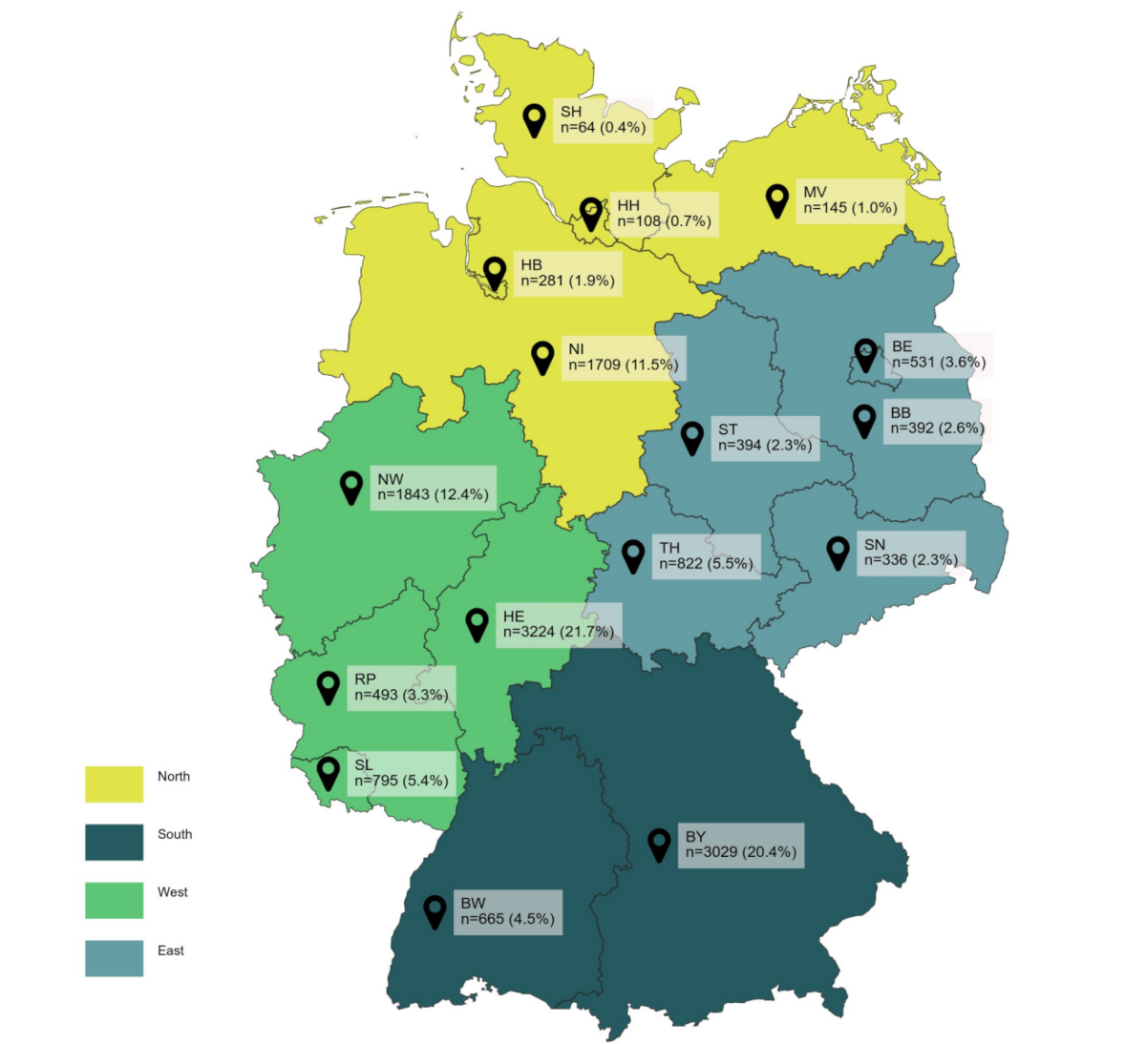
Geographical distribution of the study sample (N=14,916). BB: Brandenburg; BE: Berlin; BW: Baden-Wuerttemberg, BY: Bavaria; HB: Bremen; HE: Hesse; HH: Hamburg; MV: Mecklenburg-Western Pomerania; NI: Lower Saxony; NW: North Rhine-Westphalia; RP: Rhineland-Palatinate; SH: Schleswig-Holstein; SL=Saarland; SN: Saxony; ST: Saxony Anhalt; TH: Thuringia.

After further plausibility checks and adjustment for incorrect data, the data set contained complete questionnaires from 14,916 participants aged between 18 and 72 years (mean age: 24.3). Students from 130 universities and all 16 federal states participated (see Table 1). In terms of geographical coverage (see Figure 2), most respondents were from the west of Germany (6355/14,833, 42.8%), followed by students from the south (3694/14,833, 24.9), and almost equally from the north (2307/14,833, 15.6%) and the east (2476/14,833, 16.7%).

**Table 1:** Characteristics of the study participants (N=14,916; values are weighted).

Characteristic		Value, n (%)		
		Total	Male (n=7687, 51.5%)	Female (n=7229, 48.5%)
**Age (years; n=14,897)**				
	≤20	2640 (17.7)	1342 (17.5)	1298 (18.0)
	21-23	5495 (36.9)	2586 (33.7)	2909 (40.3)
	24-26	3567 (23.9)	1923 (25.0)	1643 (22.8)
	≥27	3195 (21.4)	1827 (23.8)	1369 (19.0)
**Study course (n=14,916)**				
	Bachelor’s degree	10,351 (69.4)	5463 (71.1)	4887 (67.6)
	Master’s degree	2796 (18.7)	1460 (19.0)	1337 (18.5)
	Other (eg, PhD)	1769 (11.9)	764 (9.9)	1005 (13.9)
**Subjective social status (n=14,913)**				
	Low	2575 (17.3)	1408 (18.3)	1168 (16.2)
	Middle	10,090 (67.7)	4974 (64.7)	5116 (70.8)
	High	2247 (15.1)	1303 (17.0)	945 (13.1)

In comparison with the whole population of German university students via the GENESIS database, some deviations could be observed. While students from Baden-Wuerttemberg (12.7% vs 4.5%) and North Rhine-Westphalia (27.5% vs 12.4%) were underrepresented in our study, our sample includes significantly more students from Bavaria (14.0% vs 20.4%) and Hesse (9.4% vs 21.7%). The gender distribution was almost balanced, with 51.5% male university students (7687/14,916) and 48.5% female students (7229/14,913). Regarding SSS, more than two-thirds of respondents reported a middle SSS (10,090/14,913, 67.7%), while 17.3% (2575/14,916) reported a low SSS and 15.1% (2247/14,913) reported a high SSS (mean SSS 6.0, SD 1.54).

**Figure 3:**
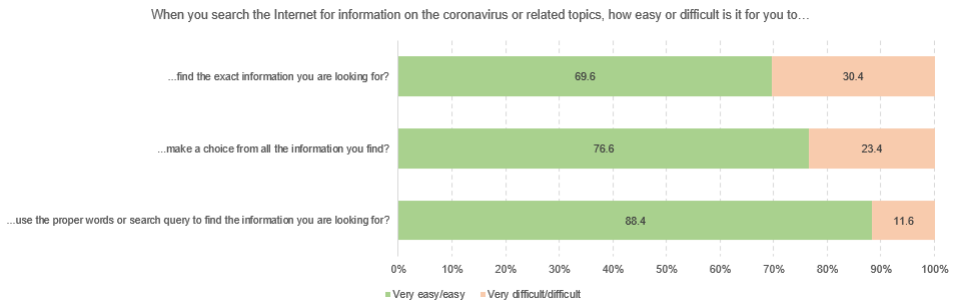
Responses to questions in the Digital Health Literacy Instrument subscale “information search” (n=14,098 to n=14,110), %.

**Figure 4:**
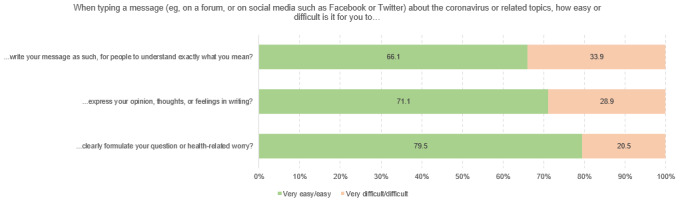
Responses to questions in the Digital Health Literacy Instrument subscale “adding self-generated content” (n=13,721 to n=13,754), %.

**Figure 5:**
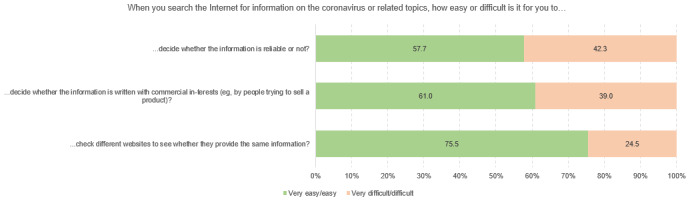
Responses to questions in the Digital Health Literacy Instrument subscale “evaluating reliability” (n=14,081 to n=14,103), %.

**Figure 6:**
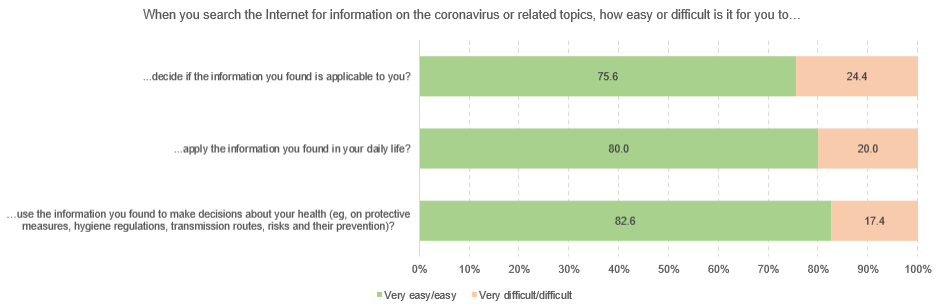
Responses to questions in the Digital Health Literacy Instrument subscale “determining relevance” (n=14,076 to n=14,092), %.

**Figure 7:**
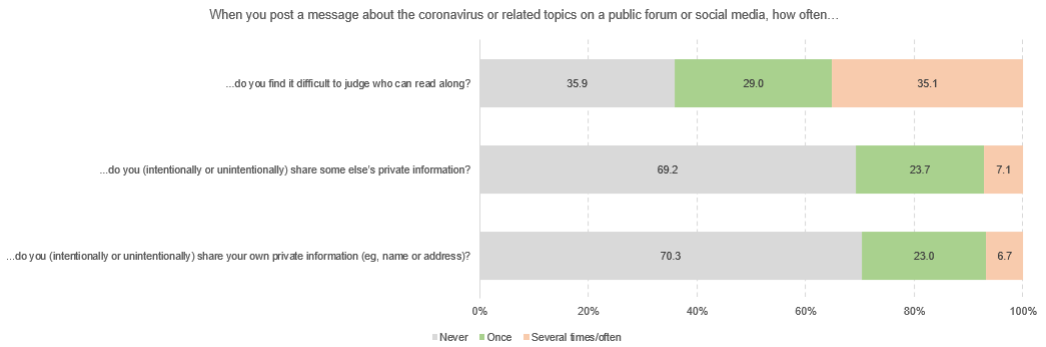
Responses to questions in the Digital Health Literacy Instrument subscale “protecting privacy” (n=13,589 to n=13,715), %.

Figures 3 to 7 show the different dimensions of digital health literacy and the percentages of student scoring. Within the “information search” subscale, university students indicated that they most frequently had problems finding the information they were looking for (4282/14,098, 30.4%), while the use of suitable words and search queries caused less difficulty (1644/14,101, 11.7%). Regarding the dimension of “adding self-generated content,” respondents reported the most difficulties in expressing their own opinion, in expressing thoughts or feelings in writing (3975/13,754, 28.9%), and in writing a message in a way that is understandable for others (4661/13,752, 33.9%). Across all dimensions, the greatest difficulties could be found in assessing the reliability of health-related information (5964/14,103, 42.3%) and the ability to determine whether the information was written with commercial interest (5489/14,097, 38.9%). The use of the found information for one’s own health-related decisions (eg, regarding protective measures, 2443/14,079, 14.4%) and the application of this information in daily life caused difficulties for approximately one-fifth of the respondents (2812/14,067, 20.0%). Finally, some heterogeneity could also be found in the items relating to the dimension of “protecting privacy.” Although approximately 35% of the respondents experienced difficulties to judge who could read messages posted on the web (4768/13,589, 35.1%), only 6.7% stated that they sometimes or often shared private information on the web (914/13,715).

Table 2 and Table 3 show the digital health literacy levels of the respondents, stratified by sociodemographic and geographic characteristics. Concerning gender, significant differences were found, with female university students showing lower digital health literacy across all subscales. However, taking the strength of the association (*V*) into account, small effect sizes could be identified only for the dimensions “information searching” (male: 2087/7219, 28.9%, female: 2711/6865, 39.5%, χ^2^_1_=175.37, *P*<.001, *V*=0.11) and “evaluating reliability” (male: 2156/5994, 36.0%, female: 2660/5630, 47.2%, χ^2^_1_=152.16, *P*<.001, *V*=0.11). All other differences were below the threshold for small effects and were hence considered trivial. When considering differentiation by age group, in all subscales, a slight tendency of increasing level of digital health literacy with increasing age was observed. However, these significant differences proved to be trivial when calculating effect sizes. The same was observed for study course, SSS, and geographical distribution. Slight differences between the groups were observed; however, the differences remained below the threshold for small effects.

**Table 2:** Digital health literacy levels of university students for the subscales of “information search” and “adding self-generated content” according to their sociodemographic and geographic characteristics.

Characteristic		Information search									Adding self-generated content							
		Limited, n (%)	Sufficient, n (%)	χ^2^ (*df*)		*P*		V		Limited, n (%)		Sufficient, n (%)	χ^2^ (*df*)		*P*		V	
**Gender**					175.37 (1)		<.001		0.11					29.77 (1)		<.001		0.05
	Male	2087 (28.9)	5132 (71.1)							2687 (38.7)		4255 (61.3)						
	Female	2711 (39.5)	4154 (60.5)							2913 (43.3)		3815 (56.7)						
**Age (years)**					17.77 (3)		<.001		0.04					78.38 (3)		<.001		0.08
	≤20	868 (35.3)	1593 (64.7)							1091 (45.7)		1295 (54.3)						
	21-23	1841 (35.5)	3340 (64.5)							2174 (43.0)		2883 (57.0)						
	24-26	1139 (33.6)	2250 (66.4)							1307 (39.8)		1975 (60.2)						
	≥27	948 (31.2)	2088 (68.8)							1020 (34.8)		1909 (65.2)						
**Study course**					26.17 (2)		<.001		0.04					98.48 (2)		<.001		0.08
	Bachelor’s degree	3431 (35.4)	6253 (64.6)							5339 (56.6)		4087 (43.4)						
	Master’s degree	848 (31.5)	1845 (68.5)							1577 (61.2)		1001 (38.8)						
	Other (eg, PhD)	519 (30.4)	1188 (69.6)							1153 (69.2)		513 (30.8)						
**Subjective social status**					11.84 (2)		.003		0.03					29.64 (2)		<.001		0.05
	Low	831 (34.5)	1580 (65.5)							1010 (43.1)		1335 (56.9)						
	Middle	3310 (34.7)	6233 (65.3)							3853 (41.6)		5409 (58.4)						
	High	655 (30.8)	1471 (69.2)							735 (35.7)		1325 (64.3)						
**Geographic location**					39.15 (3)		<.001		0.05					39.28 (3)		<.001		0.05
	North	686 (31.6)	1484 (68.4)							857 (40.6)		1255 (59.4)						
	East	786 (33.5)	1561 (66.5)							582 (37.7)		1406 (62.3)						
	West	1962 (32.7)	4036 (67.3)							2325 (39.9)		3508 (60.1)						
	South	1338 (38.3)	2154 (61.7)							1539 (45.3)		1858 (54.7)						
Total		4798 (34.1)	9286 (65.9)							5600 (41.0)		8069 (59.0)						

**Table 3:** Digital health literacy levels of university students for the subscales of “evaluating reliability” and “determining relevance” according to their sociodemographic and geographic characteristics.

Characteristic		Evaluating reliability									Determining relevance								
		Limited, n (%)	Sufficient, n (%)		χ^2^ (*df*)		*P*		V		Limited, n (%)		Sufficient, n (%)		χ^2^ (*df*)		*P*		V
**Gender**				152.16 (1)		<.001		0.11						38.10 (1)		<.001		0.05	
	Male	2156 (36.0)	3838 (64.0)								2387 (33.2)		4799 (66.8)						
	Female	2660 (47.2)	2970 (52.8)								2617 (38.2)		4232 (61.8)						
**Age (years)**				35.04 (3)		<.001		0.05						9.78 (3)		.02		0.03	
	≤20	864 (42.5)	1169 (57.5)								874 (35.7)		1571 (64.3)						
	21-23	1825 (43.5)	2373 (56.5)								1859 (36.0)		3308 (64.0)						
	24-26	1179 (42.3)	1611 (57.7)								1252 (37.1)		2124 (62.9)						
	≥27	945 (36.5)	1645 (63.5)								1013 (33.4)		2017 (66.6)						
**Study course**				23.75 (2)		<.001		0.05						9.94 (2)		.007		0.03	
	Bachelor’s degree	3434 (42.9)	4565 (57.1)								3513 (36.4)		6137 (63.6)						
	Master’s degree	846 (38.1)	1373 (61.9)								935 (34.8)		1749 (65.2)						
	Other (eg, PhD)	536 (38.1)	870 (61.9)								555 (32.6)		1146 (67.4)						
**Subjective social status**				18.69 (2)		<.001		0.04						30.11 (2)		<.001		0.05	
	Low	890 (44.0)	1133 (56.0)								928 (38.6)		1475 (61.4)						
	Middle	3258 (41.7)	4553 (58.3)								3421 (36.0)		6091 (64.0)						
	High	665 (37.2)	1121 (62.8)								655 (30.9)		1462 (69.1)						
**Geographic location**				41.08 (3)		<.001		0.06						10.43 (3)		.015		0.03	
	North	674 (38.0)	1101 (62.0)								731 (33.8)		1433 (66.2)						
	East	763 (39.1)	1187 (60.9)								855 (36.6)		1482 (63.4)						
	West	2017 (40.8)	2921 (59.2)								2086 (34.9)		3897 (65.1)						
	South	1339 (46.2)	1558 (53.8)								1300 (37.4)		2176 (62.6)						
Total		4816 (41.4)	6808 (58.6)								5004 (35.7)		9032 (64.3)						

**Figure 8:**
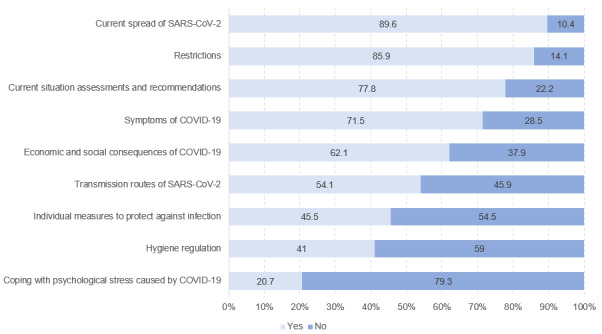
Internet search queries related to COVID-19 (n=14,111), %.

**Figure 9:**
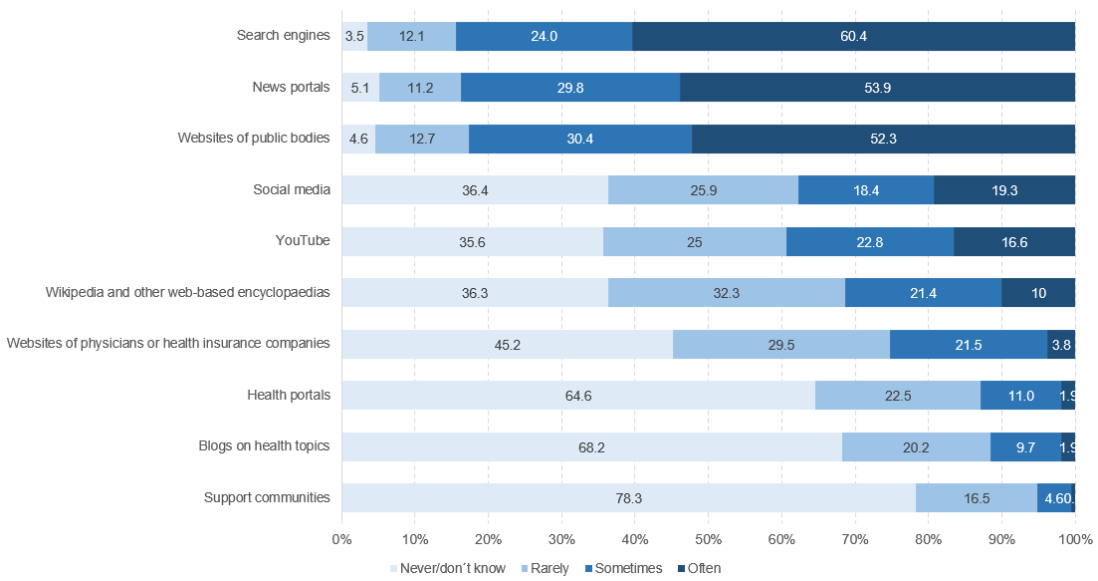
Frequency of use of internet sources for web-based health information seeking (n=14,012 to n=14,094), %.

Search engines, news portals, and websites of public bodies were most often used by the respondents as sources to search for and find information on COVID-19 and related issues (see Figure 8 and Figure 9). These sources were followed by social media platforms such as Facebook, Instagram, and Twitter, or video portals such as YouTube, with 37.6% of respondents (5302/14,092) stating that they used these media sometimes or frequently. In contrast, health-related blogs or web-based guides were used much less frequently. When stratified by sociodemographic characteristics, relevant differences could only be observed for gender. Female students were found to use social media (t_13,921_=–19.09, *P*<.001, *d*=–0.32) and health portals (t_13,463_=–14.42, *P*<.001, *d*=–0.24) more frequently than male students. In contrast, Wikipedia and other web-based encyclopedias (t_14,051_=19.19, *P*<.001, *d*=0.32), as well as YouTube (t_14,054_=18.13, *P*<.001, *d*=0.30), were more often used by male students. Regarding the topics, respondents stated that they most frequently searched for information on the current spread of SARS-CoV-2 (12,648/14,114, 89.6%) and associated restrictions (12,126/14,114, 85.9%), recommendations and assessments regarding the situation (10,975/14,114, 77.8%), and the symptoms of COVID-19 (10,089/14,114, 71.5%). Although significantly less often, one-fifth of the university students stated that they looked for information on how to cope with psychological stress caused by the COVID-19 situation (2921/14,114, 20.7%). When differentiated by sociodemographic variables, gender differences could be found, as male students searched significantly more often for information on economic and social consequences of the COVID-19 pandemic (male: 4943/7237, 68.3%, female: 3817/6878, 55.5%, χ^2^_1_=245.62, *P*<.001, *V*=.13).

Finally, digital health literacy was stratified according to web-based information-seeking behavior. No relevant differences could be found for the topics that students searched for. Regarding the sources used for the search and the handling of health-related information, significant and relevant differences emerged for the DHLI subscale “evaluating reliability.” Respondents with sufficient digital health literacy in that dimension reported using the websites of public bodies (eg, Robert Koch Institute) more frequently (t_9344_=19.44, *P*<.001, *d*=0.37). The opposite could be observed for social media (Facebook, Instagram, Twitter) (t_10,019_=–14.29, *P*<.001, *d*=–0.27) and support communities (t_9028_=–12.06, *P*<.001, *d*=–0.23), which were more frequently used by respondents who reported more difficulties in evaluating the reliability of information (see Table 4 and Table 5).

**Table 4:** Sources used for COVID-19 information search stratified by digital health literacy level for the “information search” and “adding self-generated content” subscales.

Item	Information search				Adding self-generated content						
	Limited, mean (SD)	Sufficient, mean (SD)	*P*	*d*		Limited, mean (SD)		Sufficient, mean (SD)		*P*	*d*
Search engines (eg, Google, Bing, Yahoo!)	3.44 (0.79)	3.40 (0.85)	.003	–0.05		3.43 (0.82)		3.42 (0.83)		.21	N/A^a^
Websites of public bodies (eg, Robert Koch Institute)	3.20 (0.88)	3.36 (0.85)	<.001	0.18		3.24 (0.87)		3.35 (0.85)		<.001	0.12
Wikipedia and other web-based encyclopedias	1.99 (0.96)	2.08 (0.99)	<.001	0.09		2.03 (0.98)		2.07 (0.98)		.005	0.05
Social media (eg, Facebook, Instagram, Twitter)	2.31 (1.14)	2.15 (1.12)	<.001	–0.14		2.29 (1.14)		2.17 (1.12)		<.001	–0.10
YouTube	2.19 (1.09)	2.21 (1.10)	.22	N/A		2.25 (1.10)		2.18 (1.09)		<.001	–0.07
Blogs on health topics	1.47 (0.74)	1.44 (0.75)	.056	N/A		1.46 (0.74)		1.45 (0.75)		.35	N/A
Support- communities	1.30 (0.59)	1.26 (0.56)	<.001	–0.08		1.30 (0.59)		1.27 (0.56)		.002	–0.05
Health portals	1.53 (0.77)	1.49 (0.76)	.004	–0.05		1.51 (0.77)		1.51 (0.77)		.80	N/A
Websites of physicians or health insurance companies	1.84 (0.89)	1.84 (0.89)	.72	N/A		1.82 (0.88)		1.86 (0.91)		.005	0.05
News portals (eg, newspapers, television)	3.30 (0.86)	3.34 (0.87)	.03	.04		3.32 (0.86)		3.32 (0.87)		.75	N/A

^a^N/A: not applicable due to lack of significance.

**Table 5:** Sources used for COVID-19 information search stratified by digital health literacy level for the “evaluating reliability” and “determining relevance” subscales.

Item	Evaluating reliability				Determining relevance						
	Limited, mean (SD)	Sufficient, mean (SD)	*P*	*d*		Limited, mean (SD)		Sufficient, mean (SD)		*P*	*d*
Search engines (eg, Google, Bing, Yahoo!)	3.49 (0.79)	3.36 (0.86)	<.001	–0.15		3.43 (0.82)		3.41 (0.84)		.07	N/A^a^
Websites of public bodies (eg, Robert Koch Institute)	3.13 (0.92)	3.45 (0.79)	<.001	0.37		3.22 (0.89)		3.35 (0.84)		<.001	0.16
Wikipedia and other web-based encyclopedias	2.03 (0.98)	2.08 (1.00)	.008	0.05		2.02 (0.97)		2.06 (0.99)		.01	0.04
Social media (eg, Facebook, Instagram, Twitter)	2.38 (1.15)	2.07 (1.10)	<.001	–0.27		2.28 (1.14)		2.16 (1.12)		<.001	–0.10
YouTube	2.24 (1.12)	2.17 (1.09)	.001	–0.06		2.22 (1.10)		2.19 (1.09)		.20	N/A
Blogs on health topics	1.49 (0.76)	1.42 (0.74)	<.001	–0.10		1.46 (0.74)		1.45 (0.75)		.37	N/A
Support-communities	1.36 (0.64)	1.22 (0.53)	<.001	–0.23		1.31 (0.61)		1.25 (0.55)		<.001	–0.10
Health portals	1.56 (0.80)	1.47 (0.74)	<.001	–0.12		1.52 (0.78)		1.49 (0.76)		.02	–0.04
Websites of physicians or health insurance companies	1.81 (0.88)	1.87 (0.91)	.001	0.05		1.82 (0.88)		1.85 (0.90)		.09	N/A
News portals (eg, newspapers, television)	3.30 (0.87)	3.33 (0.87)	.04	0.04		3.33 (0.84)		3.32 (0.88)		.32	N/A

^a^N/A: not applicable due to lack of significance.

## Discussion

To our knowledge, this study is the first to investigate the digital health literacy and information-seeking behaviors in university students during the COVID-19 outbreak in Germany. Nationwide and overall, university students show high levels of digital health literacy. However, one-third of all students (4282/14,098, 30.4%) reported having problems finding the correct information on a particular health-related topic. Also, almost half of all students (5964/14,103, 42.3%) had problems evaluating the reliability of web-based information, which includes difficulties in identifying commercial interests behind the information presented in the news (5489/14,097, 38.9%). Moreover, the greatest challenges were related to assessing the reliability of COVID-19–related information and to judging whether commercial interests were attached to this information. Female students reported more difficulties in searching and evaluating web-based COVID-19–related information than male students.

Although digital health literacy levels were sufficient in a large proportion of the respondents, the results must be viewed in a more differentiated way. Germany applied a very successful health communication strategy [4] based on easily understandable and easy-to-use health information regarding COVID-19 (eg, washing hands, physical distancing, wearing masks), which was of low complexity compared to other health or disease information [23]. The communication mode was primarily push-based, directing information toward people through all media and communication channels. In comparison, for noncrisis communication, people must supply themselves with information (pull communication) to a greater extent, which requires active searching for information and hence requires strong health literacy. The way in which communication was altered (push vs pull) could explain the lack of differences in digital health literacy levels in relation to socioeconomic status, which are usually found in health literacy studies [22,37]. In addition, our study reports on the state of students’ digital health literacy levels during the early stages of the so-called first wave of the pandemic, at a time when adherence to policies of measures to protect against COVID-19 was high. However, this could change in the current second wave, when people lose trust in official sources and the support for compliance with official recommendations diminishes. Declining support of public measures can already be observed in Germany. Demonstrations against restrictions are taking place, and people are demanding a return to prepandemic conditions and the reopening of the economy, all of which manifests through a refusal to apply the recommended protective measures (eg, no physical distancing, no face masks) [38].

Data protection and security is also an important issue in the context of digitalization and of the digital transformation of society. Using digital health services and communicating about health topics on the internet and on social media requires particular communication technologies to ensure user safety and user-friendliness. Our findings indicate that one-third of all students (4768/13,589, 35.1%) reported problems judging whether a third party can read their messages posted on the web. Studies on web-based data protection in the German population showed that 72% of respondents doubted the safety of the personal data they shared on the internet [39]. Moreover, 55% even believed that they had no control over what happens to their web-based data [40]. Despite its importance, we were required to exclude the subdimension “protecting privacy” from further bivariate analyses due to low reliability. When introducing the original DHLI, van der Vaart and Drossaert [20] also reported an unsatisfactory Cronbach alpha for this subscale (*α*=.57). Although this supports the validity of our study findings, it also suggests a need for further refinement (eg, by reformulating the item “…do you find it difficult to judge who can read along?“ to “…do you find it difficult to judge how the security of your private information is secured by the media provider?” to emphasize the role of protective measures taken by the media provider).

The most preferred sources to look for web-based health- and COVID-19–related information among students included search engines, news portals, and websites of public bodies, followed by social media and video portals. Favorite search topics were the current spread of COVID-19 (12,648/14,114, 89.6%), restrictions (12126/14,114, 85.9%), recommendations and risk assessments (10,975/14,114, 77.8%), and COVID-19 symptoms (10089/14,114, 71.5%). Similarly to earlier studies on population health literacy, which focus on both generic health literacy [22,41] and health literacy in relation to COVID-19 [15,23], making a judgment about the reliability of COVID-19–related information in the media and identifying potential commercial interests represent the most difficult tasks. There is also a significant positive association between having sufficient levels of digital health literacy and accessing more trustworthy and thus more reliable web-based health content. Students with higher levels of digital health literacy in the dimension of “evaluating information reliability” accessed the official websites of public bodies and agencies more often and turned less often to sources such as support communities, including forums, and social media compared to students with lower competencies in this dimension. While the ability to seek information and to produce and provide information did not show any significant differences across digital health literacy levels, students with higher abilities to determine the personal relevance of the information they obtained show similar patterns to those shown for the subscale “evaluating reliability.”

Interestingly, only one-fifth of students (2921/14,114, 20.7%) reported having searched for information related to psychological stress and the consequences of the COVID-19 pandemic on mental health. This finding is surprising, as other studies show that the COVID-19 pandemic has enormous effects on mental health [42,43], and an infodemic can trigger an epidemic of fear and anxiety [28]. On the other hand, it should be emphasized that this survey was conducted at the beginning of the first wave of the pandemic and that psychological problems became more important as the pandemic progressed. Therefore, reliable and trustworthy (mental) health information is key in this situation for citizens to act upon information and knowledge provided by governments, health authorities, and scientists, and thereby to help slow the spread of COVID-19 [14,16,44-47]. In this context, infodemiology becomes important to better understand communication patterns, information routes and content, and how they affect behaviors, attitudes, and health status [28]. Citizen behavior, however, must be facilitated by adequate government actions and policies that provide not only health information but also health, social, and economic services for citizens to cope with the situation [14]. The impact of the ongoing COVID-19 infodemic places an additional burden on web-based health information seekers. This threat amplifies the negative effects of low digital health literacy. In their representative survey of COVID-19–related health literacy during the pandemic, Okan and colleagues [23] found that 56% of the German population felt confused about the vast amount of information regarding COVID-19. Women, younger age groups, and families with children younger than 18 years in their household are significantly more affected. At the same time, people with lower income and who reside in federal states of the former East Germany were found to feel less informed than their counterparts. This ongoing study highlights that the infodemic must be acknowledged “as a meta-risk in its own right” that aggravates the current situation [23]. Therefore, this infodemic requires particular attention during the COVID-19 emergency, which includes public policy strategies aiming to address the toxic spread of misinformation and disinformation about SARS-CoV-2 and COVID-19 [14,15,23]. Moreover, producers, providers, and suppliers of health information must ensure that information is evidence-based and adheres to health literacy principles, including barrier-free and easy access, user-friendliness and ease of understanding, cultural appropriateness, and relevance for everyday public use [21,23]. Social media platforms should also counteract the spread of misinformation and disinformation about COVID-19. The fight against misinformation and disinformation should become an important issue in public policy [17,18]. As proposed by Gunter Eysenbach, the four pillars to fight an infodemic include (1) infoveillance (the monitoring of information), (2) strengthening health literacy and digital health literacy in the population, (3) applying constant knowledge refinement (eg, fact checking), and (4) adequate knowledge transfer and minimizing political and commercial influence on health information [45]. This is supported by the World Health Organization within their infodemic management framework, which suggests six policy recommendations to manage infodemics during an emergency such as the COVID-19 pandemic [46]. These recommendations include (1) basing interventions and messages on the latest evidence, (2) applying knowledge transfer and making health information easy to understand, (3) collaborating with communities to better understand their information needs, (4) analyzing information impact and cooperating with social media platforms, information suppliers, and civil society, (5) informing these actions by reliable information and adapt action based on the respective and latest narratives, and (6) further improving infodemic management by all means necessary and also through interdisciplinary research collaboration [46]. Skills to navigate digital information environments were already crucial before the COVID-19 pandemic to mitigate the effects of digital inequalities [47,48]. These skills have become even more essential during the pandemic, as the importance and use of communication technologies and media have changed massively since the outbreak of COVID-19 [14,16,45,48,49].

The most important finding of the stratified analyses is that among students with limited digital health literacy, female students reported having more problems finding the correct information and evaluating the reliability of COVID-19–related information. In Germany, women often have more care responsibilities and are generally more engaged with health issues than men [23], and they are also more active in searching the internet for health information [49,50]. This may lead them to be more critical vis-à-vis health information on COVID-19, as they have a more sensible awareness that not all information is reliable. In addition to this, a recent study showed that women are much more worried about the sheer amount of COVID-19–related information on the internet [23]. They were more concerned when they had children ≤18 years of age. Many young women are faced with difficulties and challenges when they search for and evaluate health information, especially because there is so much conflicting information on COVID-19 available on the web.

To sum up, the findings from this study raise concern and have important implications for public health. First, problems related to access to accurate and situation-specific information in the context of a public health emergency may lead to the use of invalid information, which is unhelpful or even detrimental to the causes of slowing infection rates and sustaining a successful infectious disease strategy. Second, when students access disinformation or false information and they have difficulty making judgments about the correctness of the information, they will most likely not identify that information for what it is (eg, “fake news,” commercial messaging). In turn, using the wrong information can again cause harm and impede engagement in effective health behaviors. Third, feeling safe in the digital world, especially when seeking health-related information and interacting with others about health concerns, is a critical issue. Many students expressed uncertainties regarding the safety of personal information shared on the web. These findings suggest the need to implement health education measures to strengthen students’ health literacy capacities. In addition, there is a need for more accurate public health information platforms to provide timely and evidence-based information with a view to inform individual behavior and system-level responses. Studies on health literacy in Germany conducted in 2011 [37], 2013 [41,50], and 2014 [22,41] have shown that half of the adult population, including both younger and older adults, have limited health literacy. In response, health literacy policy initiatives were launched, such as the science and civil society–led German National Action Plan on Health Literacy [51] and the Alliance for Health Literacy [52], which is led by the federal Ministry of Health. These initiatives focus on strengthening population health literacy, starting in early childhood and at school, to enable children to grow into health-literate adults. However, little progress has been made since then, and a health education curriculum that addresses health literacy is still lacking in Germany. Furthermore, people with lower education in Germany have more often lower levels of health literacy [22,37]. If students, who belong to a population group with higher education, already have difficulties with their digital health literacy, it can be assumed that people with less education are also vulnerable to having lower levels of health literacy and associated information tasks, such as finding, understanding, and evaluating COVID-19–related information on the web.

Our study has several limitations. The sample, although weighted, is not representative of all university students in Germany. We may have missed many students who use the internet less frequently and those who may have been troubled due to university closures and associated changes to their lives. The implications may not be transferable to other populations and age groups in Germany. Additionally, students in Germany are privileged in terms of educational achievement and therefore in terms of socioeconomic status compared to people seeking a tertiary education with non–degree level requirements. This survey was conducted in the early days of the first wave of the pandemic, when adherence was high; this could explain the finding that students perceived information tasks to be easy to undertake and therefore reported high levels of digital health literacy. This may not be the case in a second phase, after enduring lengthy restrictions on everyday and university life activities and rapidly emerging conflicting information on COVID-19, all of which could make judgment much more difficult. Due to the effects of the COVID-19 pandemic on physical contact and face-to-face meeting, we had to use a web-based survey in adherence to German COVID-19 policies, whereas the developers of the original questionnaire, van der Vaart and Drossaert [20], highlighted that the application of a web-based questionnaire may exclude people with weak digital competencies. Therefore, a potential bias in our sample is that it may have excluded students who use the internet to a lesser extent or those with lower digital competencies. Nevertheless, due to web-based activities related to their studies (eg, access to e-learning and university communication platforms) and given that most German universities provide their services via web-based systems, students in Germany in general represent a proportion of the population who have more intersections with the digital world, inevitably use the internet more often, and have a higher affinity to using web-based media content.

Our findings show that overall, the level of digital health literacy in relation to dealing with web-based COVID-19–related information was high. However, a significant proportion of university students still face difficulties with certain abilities to deal with information, such as finding the right information and evaluating its reliability. There is a need to strengthen the digital health literacy capacities of university students, particularly female students, using tailored interventions. Actions must also include the design of interventions to increase the quality of health information on the internet, to implement fact-checking strategies in web-based and social media, and to increase the health literacy of people who produce, supply, and provide health information and services on the web. For example, universities can provide courses on digital health literacy and health information to their staff and students and can also disseminate reliable news on COVID-19 through their web-based channels. Raising awareness among universities and education administrators might aid the emergency response, and it could also increase the health literacy responsiveness of organizations and students. The benefit of the COVID-HL survey is that it provides first-time knowledge that could help decision-makers develop policies and programs that foster healthy and protective behaviors, plan for preventive measures, and promote adherence to COVID-19 policies, on the basis of students’ needs in terms of digital health literacy. Digital health literacy will empower university students and all other population groups to take greater control in the prevention and spread of COVID-19, which in turn is likely to lead to better health outcomes.

## Appendix

Multimedia Appendix 1 Overview of scales and items.

## Abbreviations

DHLI: Digital Health Literacy Instrument
SSS: subjective social status

## References

[1] Lu R, Zhao X, Li J, Niu P, Yang B, Wu H, Wang W, Song H, Huang B, Zhu N, Bi Y, Ma X, Zhan F, Wang L, Hu T, Zhou H, Hu Z, Zhou W, Zhao L, Chen J, Meng Y, Wang J, Lin Y, Yuan J, Xie Z, Ma J, Liu WJ, Wang D, Xu W, Holmes EC, Gao GF, Wu G, Chen W, Shi W, Tan W (2020). Genomic characterisation and epidemiology of 2019 novel coronavirus: implications for virus origins and receptor binding. Lancet.

[2] (2020). World Health Organization. Novel Coronavirus (2019-nCoV) - Situation Report 13.

[3] European Centre for Disease Prevention and Control. ECDC Communicable Disease Threats Report (CDTR).

[4] Wieler L, Gottschalk R (2020). Emerging COVID-19 success story: Germany?s strong enabling environment. Our World in Data.

[5] (2020). Robert Koch Institut. Ergänzung zum Nationalen Pandemieplan – COVID-19 – neuartige Coronaviruserkrankung.

[6] Bundesministerium für Gensundheit Zusammen gegen Corona. Aktuelle Informationen zum neuartigen Coronavirus/Covid-19 Together against corona.

[7] Bundesministerium für Gesundheit. Coronavirus in Deutschland.

[8] Bundeszentrale für gesundheitliche Aufklärung. Informationen zum neuartigen Coronavirus / COVID-19 Information about the novel coronavirus/COVID-19, webpage on German.

[9] Robert Koch-Institut. COVID-19 (Coronavirus SARS-CoV-2). Webpage in German.

[10] Patienten-Information.de. Corona – verlässliche Informationen zu COVID 19.

[11] Zweites Deutsches Fernsehen. Coronavirus: Alles zu Covid-19.

[12] Norddeutscher Rundfunk. Coronavirus-Update mit Christian Drosten.

[13] Corona-Krise. Das Erste.

[14] Paakkari L, Okan O (2020). COVID-19: health literacy is an underestimated problem. Lancet Public Health.

[15] Okan O, de Sombre S, Hurrelmann K, Berens EM, Bauer U, Schaeffer D (2020). Covid-19-Gesundheitskompetenz der Bevölkerung COVID-19 based health literacy in the German population. Monitor Versorgungsforschung.

[16] Okan O, Sørensen K, Messer M (2020). COVID-19: A guide to good practice on keeping people well informed. The Conversation.

[17] Hua J, Shaw R (2020). Corona Virus (COVID-19) "Infodemic" and Emerging Issues through a Data Lens: The Case of China. Int J Environ Res Public Health.

[18] Zarocostas J (2020). How to fight an infodemic. Lancet.

[19] Sørensen K, Van den Broucke S, Fullam J, Doyle G, Pelikan J, Slonska Z, Brand H, (HLS-EU) Consortium Health Literacy Project European (2012). Health literacy and public health: a systematic review and integration of definitions and models. BMC Public Health.

[20] van der Vaart Rosalie, Drossaert C (2017). Development of the Digital Health Literacy Instrument: Measuring a Broad Spectrum of Health 1.0 and Health 2.0 Skills. J Med Internet Res.

[21] Sørensen K (2020). Covid-19: Digital health literacy is a key to saving time, costs and lives. ICT & health.

[22] Schaeffer D, Berens E, Vogt D (2017). Health Literacy in the German Population. Dtsch Arztebl Int.

[23] Okan O, Bollweg TM, Berens E, Hurrelmann K, Bauer U, Schaeffer D (2020). Coronavirus-Related Health Literacy: A Cross-Sectional Study in Adults during the COVID-19 Infodemic in Germany. Int J Environ Res Public Health.

[24] Van den Broucke S (2020). Why health promotion matters to the COVID-19 pandemic, and vice versa. Health Promot Int.

[25] Horgan Á, Sweeney J (2012). University students' online habits and their use of the Internet for health information. Comput Inform Nurs.

[26] Dadaczynski K, Okan O, Messer M, Rathmann K (2020). Digitale Gesundheitskompetenz von Studierenden in Deutschland während der Corona-Pandemie. Ergebnisse einer bundesweiten Online-Befragung. Hochschule Fulda.

[27] Nguyen HT, Do BN, Pham KM, Kim GB, Dam HTB, Nguyen TT, Nguyen TTP, Nguyen YH, Sørensen Kristine, Pleasant A, Duong TV (2020). Fear of COVID-19 Scale-Associations of Its Scores with Health Literacy and Health-Related Behaviors among Medical Students. Int J Environ Res Public Health.

[28] Eysenbach G (2011). Infodemiology and infoveillance tracking online health information and cyberbehavior for public health. Am J Prev Med.

[29] (2019). Statistische Daten zu Studienangeboten an Hochschulen in Deutschland. Studiengänge, Studierende, Absolventinnen und Absolventen. Wintersemester 2019. Hochschulrektorenkonferenz.

[30] Robert Koch-Institut: COVID-19-Dashboard. Webpage in German. Robert Koch-Institut.

[31] (2019). EFS Survey, Version EFS Winter 2018. Questback GmbH.

[32] Hoebel J, Müters S, Kuntz B, Lange C, Lampert T (2015). Measuring subjective social status in health research with a German version of the MacArthur Scale. Article in German. Bundesgesundheitsblatt Gesundheitsforschung Gesundheitsschutz.

[33] Schricker J, Rathmann K, Dadaczynski K (2019). Soziale Unterschiede in der Gesundheitskompetenz von Studierenden: Ergebnisse einer Online-Studie an der Technischen Universität Dortmund. Präv Gesundheitsf.

[34] Marstedt G (2018). Das Internet: Auch Ihr Ratgeber für Gesundheitsfragen? Bevölkerungsumfrage zur Suche von Gesundheitsinformationen im Internet und zur Reaktion der Ärzte.

[35] Die Datenbank des Statistischen Bundesamtes. Federal Statistical Office of Germany.

[36] Cohen J (2013). Statistical Power Analysis for the Behavioral Sciences.

[37] Sørensen K, Pelikan JM, Röthlin F, Ganahl K, Slonska Z, Doyle G, Fullam J, Kondilis B, Agrafiotis D, Uiters E, Falcon M, Mensing M, Tchamov K, van den Broucke S, Brand H, HLS-EU Consortium (2015). Health literacy in Europe: comparative results of the European health literacy survey (HLS-EU). Eur J Public Health.

[38] Sueddeutsche Z (2020). Berlin: 20.000 Menschen demonstrieren gegen Corona-Auflagen. Süddeutsche Zeitung.

[39] Nagler K, Schröder D, Schilling A, Weber T (2020). DsiN-Sicherheitsindex 2020. Studie von Deutschland sicher im Netz.

[40] Studie zu Datenschutz: Mehrheit der Deutschen zweifelt an Datensicherheit. Sinus Institut.

[41] Berens E, Vogt D, Messer M, Hurrelmann K, Schaeffer D (2016). Health literacy among different age groups in Germany: results of a cross-sectional survey. BMC Public Health.

[42] Ahorsu DK, Lin C, Imani V, Saffari M, Griffiths MD, Pakpour AH (2020). The Fear of COVID-19 Scale: Development and Initial Validation. Int J Ment Health Addict.

[43] Satici B, Gocet-Tekin E, Deniz ME, Satici SA (2020). Adaptation of the Fear of COVID-19 Scale: Its Association with Psychological Distress and Life Satisfaction in Turkey. Int J Ment Health Addict.

[44] Sentell T, Vamos S, Okan O (2020). Interdisciplinary Perspectives on Health Literacy Research Around the World: More Important Than Ever in a Time of COVID-19. Int J Environ Res Public Health.

[45] Eysenbach G (2020). How to Fight an Infodemic: The Four Pillars of Infodemic Management. J Med Internet Res.

[46] Tangcharoensathien V, Calleja N, Nguyen T, Purnat T, D'Agostino M, Garcia-Saiso S, Landry M, Rashidian A, Hamilton C, AbdAllah A, Ghiga I, Hill A, Hougendobler D, van Andel J, Nunn M, Brooks I, Sacco PL, De Domenico M, Mai P, Gruzd A, Alaphilippe A, Briand S (2020). Framework for Managing the COVID-19 Infodemic: Methods and Results of an Online, Crowdsourced WHO Technical Consultation. J Med Internet Res.

[47] Beaunoyer E, Dupéré S, Guitton MJ (2020). COVID-19 and digital inequalities: Reciprocal impacts and mitigation strategies. Comput Human Behav.

[48] Guitton MJ (2020). Cyberpsychology research and COVID-19. Comput Human Behav.

[49] Bidmon S, Terlutter R (2015). Gender Differences in Searching for Health Information on the Internet and the Virtual Patient-Physician Relationship in Germany: Exploratory Results on How Men and Women Differ and Why. J Med Internet Res.

[50] Jordan S, Hoebel J (2015). [Health literacy of adults in Germany: Findings from the German Health Update (GEDA) study]. Bundesgesundheitsblatt Gesundheitsforschung Gesundheitsschutz.

[51] Schaeffer D, Hurrelmann K, Bauer U, Kolpatzik K (2018). National Action Plan Health Literacy. Promoting Health Literacy in Germany.

[52] Bundesministerium für Gesundheit. Allianz für Gesundheitskompetenz.

