# Supplementary material for: Digital Health Literacy and Web-Based Information-Seeking Behaviors of University Students in Germany During the COVID-19 Pandemic: Cross-sectional Survey Study
Source: J Med Internet Res. 2021 Jan 15;23(1):e24097. doi: 10.2196/24097 (PMC7813561; doi:10.2196/24097)
Supplement: Multimedia Appendix 1 [file jmir_v23i1e24097_app1.docx]

**Multimedia Appendix** Overview of scales and items**.**

This appendix contains all items and scales that are used for this paper.

1. **Sociodemographic information**
   1. Gender
   2. Age
   3. Study programme
   4. Subjective Social Status
2. **Digital Health Literacy**
   1. DHLI: information search
   2. DHLI: self-generated content
   3. DHLI: evaluating reliability
   4. DHLI: determine relevance
3. **Online information seeking behaviour**
   1. Online sources used in the context of COVID-19
   2. Topics searched for in the context of COVID-19

The whole questionnaire is available on request from the first authors.

| **Sociodemographic Information** |
| --- |

| 1. | **Please indicate your sex.** | | | | | |  |
| --- | --- | --- | --- | --- | --- | --- | --- |
|  | 🞏 | Female | 🞏 | Male | 🞏 | Diverse | |

| 2. | **How old are you?** |  |
| --- | --- | --- |
|  | I am __________ years old. | |

| 3. | **Are you studying in a Bachelor's or Master's programme?** | | | | | |  |
| --- | --- | --- | --- | --- | --- | --- | --- |
|  | 🞏 | Bachelor | 🞏 | Master | 🞏 | Other (eg. PhD) | |

| 4. | **Please think of a ladder as representing where people stand in Germany.** | | | |  |
| --- | --- | --- | --- | --- | --- |
|  | - At the top of the ladder are the people who are the best off – those who have the most money, the most education, and the most respected jobs. - At the bottom are the people who are the worst off – those who have the least money, least education, the least respected jobs, or no job.   The higher up you are on this ladder, the closer you are to the people at the very top; the lower you are, the closer you are to the people at the very bottom.  **Where would you place yourself on this ladder?**  Please mark a field from 1-10 where you think you stand at this time in your life relative to other people in Germany. |  | 10 | 🞏 |  |
|  |  |  | 9 | 🞏 |  |
|  |  |  | 8 | 🞏 |  |
|  |  |  | 7 | 🞏 |  |
|  |  |  | 6 | 🞏 |  |
|  |  |  | 5 | 🞏 |  |
|  |  |  | 4 | 🞏 |  |
|  |  |  | 3 | 🞏 |  |
|  |  |  | 2 | 🞏 |  |
|  |  |  | 1 | 🞏 |  |

| **Digital Health Literacy** |
| --- |

| 5. | **When you search the Internet for information on the coronavirus or related topics, how easy or difficult is it for you to…** | | | | | |  |
| --- | --- | --- | --- | --- | --- | --- | --- |
|  |  |  | Very  easy | Easy | Difficult | Very  difficult | |
|  | ... | make a choice from all the information you find? | 🞏 | 🞏 | 🞏 | 🞏 | |
|  | ... | use the proper words or search query to find the information you are looking for? | 🞏 | 🞏 | 🞏 | 🞏 | |
|  | … | find the exact information you are looking for? | 🞏 | 🞏 | 🞏 | 🞏 | |

| 6. | **When typing a message (eg, on a forum, or on social media such as Facebook or Twitter) about the coronavirus or related topics, how easy or difficult is it for you to…** | | | | | |  |
| --- | --- | --- | --- | --- | --- | --- | --- |
|  |  |  | Very  easy | Easy | Difficult | Very  difficult | |
|  | ... | clearly formulate your question or health-related worry? | 🞏 | 🞏 | 🞏 | 🞏 | |
|  | ... | express your opinion, thoughts, or feelings in writing? | 🞏 | 🞏 | 🞏 | 🞏 | |
|  | … | write your message as such, for people to understand exactly what you mean? | 🞏 | 🞏 | 🞏 | 🞏 | |

| 7. | **When you search the Internet for information on the coronavirus or related topics, how easy or difficult is it for you to…** | | | | | |  |
| --- | --- | --- | --- | --- | --- | --- | --- |
|  |  |  | Very  easy | Easy | Difficult | Very  difficult | |
|  | ... | decide whether the information is reliable or not? | 🞏 | 🞏 | 🞏 | 🞏 | |
|  | ... | decide whether the information is written with commercial interests (eg, by people trying to sell a product)? | 🞏 | 🞏 | 🞏 | 🞏 | |
|  | … | check different websites to see whether they provide the same information? | 🞏 | 🞏 | 🞏 | 🞏 | |

| 8. | **When you search the Internet for information on the coronavirus or related topics, how easy or difficult is it for you to…** | | | | | |  |
| --- | --- | --- | --- | --- | --- | --- | --- |
|  |  |  | Very  easy | Easy | Difficult | Very  difficult | |
|  | ... | decide if the information you found is applicable to you? | 🞏 | 🞏 | 🞏 | 🞏 | |
|  | ... | apply the information you found in your daily life? | 🞏 | 🞏 | 🞏 | 🞏 | |
|  | … | Use the information you found to make decisions about your health (eg, on protective measures, hygiene regulations, transmission routes, risks and their prevention)? | 🞏 | 🞏 | 🞏 | 🞏 | |

| 9. | **When you post a message about the coronavirus or related topics on a public forum or social media, how often…** | | | | | | | | |  |
| --- | --- | --- | --- | --- | --- | --- | --- | --- | --- | --- |
|  |  |  | Never | Once | | Several time | | | Often | |
|  | ... | do you find it difficult to judge who can read along? | 🞏 | | 🞏 | | 🞏 | 🞏 | | |
|  | ... | do you (intentionally or unintentionally) share your own private information (eg, name or address)? | 🞏 | | 🞏 | | 🞏 | 🞏 | | |
|  | ... | do you (intentionally or unintentionally) share some else’s private information? | 🞏 | | 🞏 | | 🞏 | 🞏 | | |

| **Online information seeking behaviour** |
| --- |

| **10.** | **Now various possibilities are mentioned how to get information about the coronavirus and related topics on the Internet. Please indicate how often you currently use these sources.** | | | | | |  |
| --- | --- | --- | --- | --- | --- | --- | --- |
|  |  | Often | Sometimes | Rarely | Never | Don´t know | |
|  | Search engines (eg, Google, Bing, Yahoo!) | 🞏 | 🞏 | 🞏 | 🞏 | 🞏 | |
|  | Websites of public bodies (eg, RKI, BZgA, ministries of health) | 🞏 | 🞏 | 🞏 | 🞏 | 🞏 | |
|  | Wikipedia and other web-based encyclopedias | 🞏 | 🞏 | 🞏 | 🞏 | 🞏 | |
|  | Social media (eg, Facebook, Instagram, Twitter) | 🞏 | 🞏 | 🞏 | 🞏 | 🞏 | |
|  | YouTube | 🞏 | 🞏 | 🞏 | 🞏 | 🞏 | |
|  | Blogs on health topics | 🞏 | 🞏 | 🞏 | 🞏 | 🞏 | |
|  | Support-communities (eg, gutefrage.de) | 🞏 | 🞏 | 🞏 | 🞏 | 🞏 | |
|  | Health portals (eg, Onmeda, Netdoktor) | 🞏 | 🞏 | 🞏 | 🞏 | 🞏 | |
|  | Websites of physicians or health insurance companies | 🞏 | 🞏 | 🞏 | 🞏 | 🞏 | |
|  | News portals (eg, of newspapers, TV stations) | 🞏 | 🞏 | 🞏 | 🞏 | 🞏 | |

| 11. | **Please indicate the specific topics you are searching for in the context of the coronavirus.**  You can select multiple response options if necessary. | |  |
| --- | --- | --- | --- |
|  | 🞏 | Current spread of the SARS-CoV-2 (eg, number of infected cases) | |
|  | 🞏 | Transmission routes of SARS-CoV-2 | |
|  | 🞏 | Symptoms of COVID-19 | |
|  | 🞏 | Individual measures to protect against infection (eg, hand washing tips) | |
|  | 🞏 | Hygiene regulations (eg, disinfection & cleaning) | |
|  | 🞏 | Current situation assessments and recommendations (eg. RKI) | |
|  | 🞏 | Restrictions (eg, exit restrictions, stay-at-home orders) | |
|  | 🞏 | Economic and social consequences of COVID-19 | |
|  | 🞏 | Coping with psychological stress caused by COVID-19 | |
|  | 🞏 | Others, namely: ______________________________________________________ | |

**You've done it! Thank you very much for your participation.**

The results of this study will be published on the websites of the study team (Public Health Centre Fulda, Interdisciplinary Centre for Health Literacy Research

of Bielefeld University). You can now close the page.
